# Face adaptation aftereffects reveal anterior medial temporal cortex role in high level category representation

**DOI:** 10.1016/j.neuroimage.2007.04.057

**Published:** 2007-08-01

**Authors:** N. Furl, N.J. van Rijsbergen, A. Treves, R.J. Dolan

**Affiliations:** a Wellcome Department of Imaging Neuroscience, Institute of Neurology, University College London, 12 Queen Square, London, WC1N 3BG, UK; b Cognitive Neuroscience Sector, Scuola Internazionale Superiore di Studi Avanzati (SISSA), via Beirut 2/4, 34104 Trieste, Italy

## Abstract

Previous studies have shown reductions of the functional magnetic resonance imaging (fMRI) signal in response to repetition of specific visual stimuli. We examined how adaptation affects the neural responses associated with categorization behavior, using face adaptation aftereffects. Adaptation to a given facial category biases categorization towards non-adapted facial categories in response to presentation of ambiguous morphs. We explored a hypothesis, posed by recent psychophysical studies, that these adaptation-induced categorizations are mediated by activity in relatively advanced stages within the occipitotemporal visual processing stream. Replicating these studies, we find that adaptation to a facial expression heightens perception of non-adapted expressions. Using comparable behavioral methods, we also show that adaptation to a specific identity heightens perception of a second identity in morph faces. We show both expression and identity effects to be associated with heightened anterior medial temporal lobe activity, specifically when perceiving the non-adapted category. These regions, incorporating bilateral anterior ventral rhinal cortices, perirhinal cortex and left anterior hippocampus are regions previously implicated in high-level visual perception. These categorization effects were not evident in fusiform or occipital gyri, although activity in these regions was reduced to repeated faces. The findings suggest that adaptation-induced perception is mediated by activity in regions downstream to those showing reductions due to stimulus repetition.

## Introduction

Although faces are highly similar visual objects, which may be encountered under a variety of circumstances (e.g., illumination, pose, etc.), humans possess an extraordinary ability to discriminate among their component categories such as identity and expression. A number of functional magnetic resonance imaging (fMRI) studies have studied category selectivity in regions in ventral temporal cortex by contrasting conditions in which stimulus information is repeated against conditions in which the stimulus information changes (Grill-Spector et al., 2006). This research raises unresolved questions concerning how and whether repetitive experience with a visual category (adaptation) influences overt perceptual categorization behavior. The approach we used here relies on adaptation to induce different perceptions, while holding the stimulus set constant. In such experiments using adaptation aftereffects, often referred to as “the psychologist's microelectrode” (Frisby, 1980), prolonged or repeated exposure to a visual attribute increases perception of a non-adapted or “opposing” attribute in response to subsequently presented ambiguous stimuli.

Historically, these studies focused on simple manipulations such as position (Köhler and Wallach, 1944), orientation (Gibson and Radner, 1937), curvature (Gibson, 1933), spatial frequency (Blakemore and Sutton, 1969), motion direction (Huk et al., 2001) and color/orientation contingencies (McCollough, 1965). More recent experiments show that adaptation can also affect perception of natural face categories including facial expression (Hsu and Young, 2004; Webster et al., 2004), identity (Leopold et al., 2001; Leopold et al., 2005), race and gender (Webster et al., 2004). Adaptation has also been shown to affect perception of viewpoint (Fang and He, 2005) and normality of distorted faces (Yamashita et al., 2005).

Collectively, these psychophysics studies pose a hypothesis that adaptation-induced categorization effects are associated with high level neural representations that are tuned to global stimulus categories, rather than simple visual features. In line with this idea, adaptation to synthetic faces which differ geometrically, but do not vary in simple visual attributes, is sufficient to produce face identity aftereffects, suggesting involvement of neural populations coding complex attributes (Anderson and Wilson, 2005). Moreover, face aftereffects are to some extent invariant to changes in simple visual features of the adapting and test stimuli including contrast, color, size (Yamashita et al., 2005), orientation (Watson and Clifford, 2003) and translation (Fang and He, 2005; Leopold et al., 2001).

Concomitantly, face aftereffects show high-level domain-specificity. In expression (Hsu and Young, 2004) and face-distortion (Yamashita et al., 2005) adaptation experiments, opposite aftereffects can be simultaneously induced in two identities, suggesting that adaptation can differentially access high-level neural populations which code for individual identities. Aftereffects also show other types of contingency on the global category of the adapting and test stimuli: gender aftereffects can be induced for face or hand categories without transfer (Kovács et al., 2006), and viewpoint aftereffects do not transfer between face, car and wire-like object categories (Fang and He, 2005), suggesting that these aftereffects are associated with face-specific neural populations. Moradi and colleagues (Moradi et al., 2005) have shown that suppression of adaptation using binocular rivalry disrupted the face identity aftereffect while preserving the low-level orientation aftereffect, suggesting that face aftereffects (but not orientation aftereffects) are mediated by a late visual processing stage, subsequent to the resolution of rivalry. Thus, face aftereffects may be mediated by plasticity within visual representations that are non-retinotopic, are relatively invariant to simple visual features and exhibit specific tuning for global visual categories (Leopold et al., 2001).

Plasticity of high-level global category representations might reflect influences at relatively advanced levels of processing in anterior regions of the occipitotemporal visual hierarchy. Although prior studies show effects of face repetition in regions such as fusiform gyrus, it is unknown whether these and/or other ventral stream regions directly play a role in perceptual categorization effects. In the present study, we used fMRI to identify visual systems mediating influences of adaptation on perceptual categorizations of facial identity and expression. Behaviorally, we elicited expression and identity aftereffects using comparable methods. For expression adaptation, adaptation to a given expression increased perception of the non-adapted expression in test faces morphed between the two expressions. We similarly found that adaptation to a given identity increased perception of the non-adapted identity in faces morphed between the two identities. During categorizations associated with perceptual bias, we observed modulation of activity in anterior medial temporal structures, including perirhinal cortex and anterior hippocampus. These categorization effects were not also observed in areas such as occipital cortex and fusiform gyrus, although these regions showed repetition suppression. We conclude that adaptation influences categorization behavior implemented within relatively late stages of a ventral visual processing pathway including anterior medial temporal lobe structures.

## Materials and methods

### Subjects

Sixteen volunteers participated in the experiment. Of these, data for four subjects were excluded: one due to excessive head motion and three due to equipment malfunction resulting in loss of behavioral data. Of the remaining 12 subjects included in the analysis (ages ranged from 19 to 26), nine were female and all had normal or corrected-to-normal vision. Informed consent was obtained in accordance with procedures approved by The Joint Ethics Committee of The National Hospital for Neurology and Neurosurgery and The Institute of Neurology, London.

### Stimuli

**Fig. 1:**
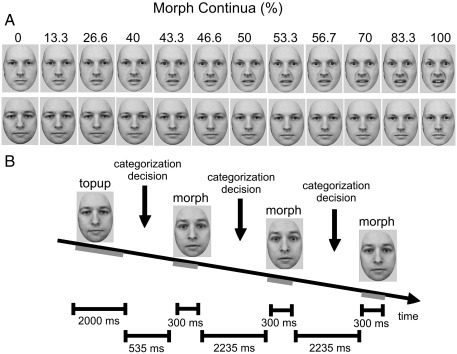
Stimuli and procedures. (A) Examples of morph images used during experiment, including a fearful/neutral continuum (top) and an identity continuum (bottom). Numbers represent the percentage in each morph image of fearful expression (top) and strength of the identity Jim (bottom). (B) Procedure for categorization trials. Following an adaptation period, subjects categorized a series of images, including top-up exposures to the adapting stimulus, each followed by one, two or three presentations of test faces, sampled from the morph continuum. In this example, the adapting stimulus is Bob, with a neutral expression, and faces from the Bob fearful-neutral continuum are categorized as “fearful” or “neutral.”

Stimuli consisted of images of two male facial identities (i.e., labeled “Jim” and “Bob”), selected from the KDEF database (Lundqvist et al., 1998; The Karolinska Directed Emotional Faces, Department of Clinical Neuroscience, Psychology Section, Karolinska Institute). For each of the two identities, we selected three images, depicting neutral, fearful and happy expressions. Using MATLAB (Mathworks, Natick, MA), face images were converted to greyscale and placed within a grey oval frame (thereby occluding hair, clothing, etc.). Then, using landmark-based morphing software (SqirlzMorph 1.2), we constructed seven symmetric morph continua (Fig. 1A), each comprising 30 equally spaced intermediate images (in steps of 3.3%). The neutral expression images were morphed with the happy and fearful expression images (i.e., happy/neutral and fearful/neutral continua) separately for each of the two identities, producing four continua spanning neutral and emotive expressions. Three additional continua were created, spanning the two identities (i.e., Jim/Bob continua): one between the neutral expression images, one between the fearful expression images, and one between the happy expression images.

### fMRI experiment

After administering a short practice session, intended as a familiarization with the experimental procedures and the two identities (i.e., “Jim” and “Bob”), we placed subjects in the scanner where they performed the main experiment. All behavioral measures were collected during the scanning session. Adaptation conditions were organized into fourteen blocks, two of which were carried out in each of seven scanning sessions. The order of the blocks over sessions was pseudo-randomized. Each block could have one of three instructions: categorize each face as fearful or neutral (four blocks), as happy or neutral (four blocks), or as “Bob” or “Jim” (six blocks). Each block commenced with a 10-s instruction screen, followed by the adaptation period, during which subjects viewed (and categorized) 20 presentations of one endpoint of a morph continuum (800 ms in duration). Each presentation was followed immediately by a 50-ms greyscale pattern mask and then by a 400-ms fixation cross. Adaptation to this endpoint was followed by a categorization period, during which subjects viewed 72 face images interspersed with 16 null trials (in which only a fixation cross was shown). All trials, including null trials, were 2535 ms in duration. Twenty-four of the face trials were top-up trials, which served to refresh the effects of adaptation throughout the categorization period via continued repetition of the adapting image. During a top-up trial, the adapting image was shown for 2 s. Each top-up trial was then followed by one, two or three images sampled from the morph continuum, each shown for 300 ms (Fig. 1B). The variable number of morphs following each topup was introduced to ensure the onsets of morphs were jittered relative to the onsets of topups such that the hemodynamic responses to each would not be correlated in time. Throughout a block, subjects viewed and categorized 48 morph images. We included a range of morph levels, to prevent repetitive categorization of one identical image. However, as we aimed to evaluate effects of adaptation on responses to ambiguous images, we over-sampled the midrange of each continuum, where effects of adaptation were expected to be the largest. Specifically, six morph levels straddling the midrange (40%, 43.3%, 46.6%, 50%, 53.3%, 56.7%) were each presented six times per block. Four additional morph levels, nearer to the endpoints were also included, but were presented only three times each (13.3%, 26.6%, 70%, 83.3%). Blocks were separated by a baseline period, during which subjects viewed a fixation point for 16 s.

### fMRI scanning

A Siemens Allegra 3T system (Siemens, Erlangen, Germany) was used to acquire T2*-weighted echoplanar functional brain volumes, using a standard head coil. A total of 1932 volumes were collected. Each volume consisted of 32 slices (tilted 10° relative to axial), was collected once every 2.08 s at an in-plane resolution of 3 × 3 mm, had 2 mm slice thickness, 1 mm slice gap with TE = 30 ms and a flip angle of 90°. The first five volumes collected from each session were discarded to allow for T1 equilibrium effects. Functional images were reconstructed using a trajectory-based reconstruction to minimize image artefacts (e.g., ghosting and distortion).

### Data preprocessing

fMRI data were preprocessed using SPM2 (Wellcome Department of Imaging Neuroscience, London; http://www.fil.ion.ucl.ac.uk/spm/). After temporal realignment of the time series to acquisition of the middle slice, data were spatially realigned and unwarped. Next, images were spatially normalized to a standard Montreal Neurological Institute (MNI) template image in Talairach space and smoothed to a 9-mm full-width half-maximum Gaussian kernel.

### Data analysis

Behavioral data were analyzed using SPSS 11.0 and fMRI data were analyzed in MATLAB 6.5.1 using a two-level model, as implemented in SPM2. At the first level, fixed effects time series models were constructed for each subject using the mass univariate approach. Data were proportionately scaled, autocorrelations were modeled using an AR(1) process and the data were high-pass filtered at 1/128 Hz. Each fMRI time series was modeled using a general linear model design matrix including regressors derived via the convolution of a canonical hemodynamic response model with delta functions representing the onsets of each event type. For each block, regressors were created to model hemodynamic responsivity to the instruction phase, the adaptation period, top-up events and morph events. Morph events were subdivided into separate regressors according to whether or not the subject perceived a category matching the adapted category. Although we included a range of morph levels in the experiment, our aim was to assess effects of adaptation on responses to category-ambiguous images only and to remove potential effects of morph levels from hypothesis testing. Thus, we further subdivided the morph level events into two more sets of regressors: one set collapsing over the closely-spaced intermediate morph levels, and another collapsing over extreme morph levels (13.3% and 83.3%). Only the former set of regressors was used for computing contrasts. Although the use of a narrow range of morph levels is aimed at minimizing correlations between the fMRI signal and morph level, we nevertheless included morph level as a parametric nuisance variable, to control for any residual covariation (these nuisance regressors showed no effects of morph level at *P* < 0.001, uncorrected). For each subject, we examined interaction effects between adapted category and perceived category (separately for expression and identity categorization blocks), by computing linear contrast images for each subject at the fixed effects level, and then entering these into one sample *t*-tests (treating subject as a random factor), to afford statistical hypothesis testing at the population level. We focus our conclusions primarily on results which survive a Gaussian random field-based family-wise error (FWE) whole brain correction for multiple comparisons and surpassed a cluster threshold of 10 voxels of 2 mm^3^ in size. The sizes of clusters (in voxels) were assessed at *P* < 0.001, uncorrected.

In addition to evaluating responses to morph faces, we analyzed responses to blocks of repeated faces during the initial adaptation period. At the fixed effects, individual subject level we computed contrasts comparing (a) initial adaptation periods against fixation baseline; (b) emotional versus neutral adaptation periods and (c) the interaction of fearful versus neutral and happy versus neutral contrasts. These contrasts were then each evaluated at the second, random effects, level using one-sample *t*-tests. We also wished to observe effects of repetition for those regions which showed responses significantly greater than for the fixation baseline. Thus, for the voxel within each of these regions which showed the peak activation relative to baseline, we recovered the time course over the block of repeated faces by averaging across subjects the results of a finite impulse response model with 3 s bins.

## Results

### Behavioral results

**Fig. 2:**
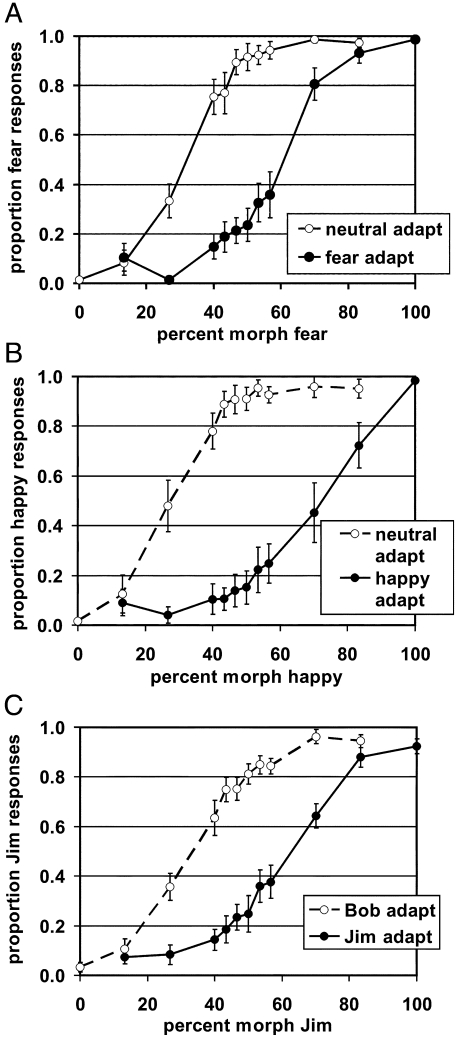
Behavioral results. Mean proportion faces categorized as (A) “fearful,” (B) “happy,” and (C) “Jim” plotted as a function of morph level and adapted category. Vertical bars represent standard errors.

Fig. 2 shows psychometric data for fearful/neutral (Fig. 2A), happy/neutral (Fig. 2B) and identity (Fig. 2C) blocks. For both expression categorization tasks, subjects' responses transitioned between “neutral” and “emotive” over the morph continua, corresponding to the boundary between expression categories (Young et al., 1997). As in previous studies (Hsu and Young, 2004; Webster et al., 2004), adaptation to a given expression category shifted the position of this category boundary along the morph continua in the direction of the adapted expression, resulting in increased perception of the non-adapted category. Fearful/neutral and happy/neutral blocks evinced similar patterns. For identity categorization, a similar transition occurred between “Bob” and “Jim” responses across the identity morph continua, corresponding to the boundary between the two identity categories. Similar to the expression results, adaptation to a given identity shifted the category boundary away from the adapted identity, increasing perception of the non-adapted identity in the morph faces. Both expression and identity categorization show significant two-way interactions between adaptation condition and morph level using ANOVAs with Greenhouse–Geisser correction for non-sphericity (expression categorization blocks: *F*(3.845,42.3) = 38.6, *P* < 0.001; identity categorization blocks: *F*(2.1,39.3) = 14.14.8, *P* < 0.001).

### fMRI responses to morph faces

**Fig. 3:**
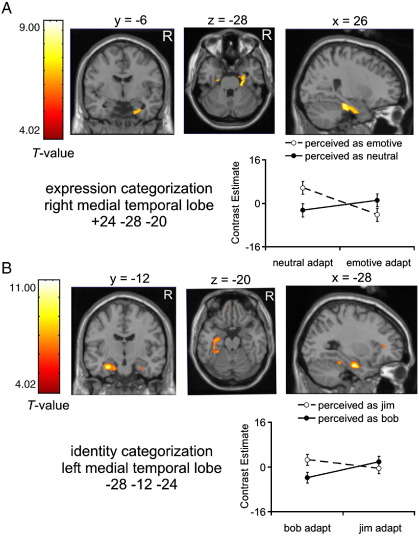
Medial temporal lobe effects for morph faces. Statistical parametric map of *T*-values showing adapt × perception interaction effects for the right medial temporal cluster (*P* < 0.001, uncorrected; overlaid on single subject T1 image) for expression categorization (A) and the left medial temporal cluster for identity categorization. For each region of interest, shown is the pattern of contrast estimates with 90% confidence intervals.

We next tested for effects of adaptation in relation to category perception. For expression categorization, our aim was to identify regions where the adapted category modulated activity underlying the subjects' perception of expression in ambiguous morph images. That is, we tested for the interaction of adapted expression and perceived expression, collapsing together observations from fearful/neutral and happy/neutral categorization blocks to maximize statistical power.^1^ Critical effects were observed in this interaction in bilateral anterior medial temporal structures where adaptation to a given expression enhanced activity during perception of the non-adapted expression. In the right hemisphere (Fig. 3A), a cluster of 287 voxels extended in a rostral–caudal axis along the rhinal sulcus, just medial to the fusiform gyrus, and ventral to the hippocampus, encompassing parts of the perirhinal and entorhinal cortices, with the peak effect in the perirhinal cortex (MNI coordinate of peak: 24 − 28 − 20). This cluster survived FWE correction for the whole brain (*P* = 0.001). Fig. 3A shows the pattern of contrast estimates associated with this interaction. In the left hemisphere, the same contrast revealed an anterior medial temporal cluster of 23 voxels with a peak effect located in perirhinal cortex (MNI coordinate of peak: − 22 − 16 − 28). The left hemisphere cluster was significant at *P* < 0.001, uncorrected, but did not survive FWE correction. Interestingly, no effects were observed in any voxels within the fusiform gyrus or amygdala (regions which are typically face-responsive).

**Fig. 4:**
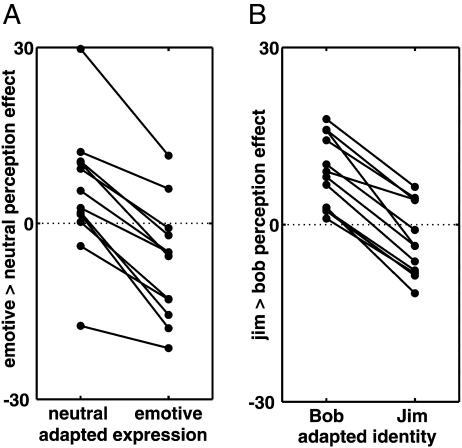
Differential effects of perception in the medial temporal lobe of individual subjects. (A) Individual subject effects at right medial temporal effect shown in Fig. 3. The contrast comparing emotive versus neutral perception is plotted separately for neutral and emotive adaptation. For most subjects, this difference is positive after neutral adaptation, but is negative after emotive adaptation. (B) Individual subject effects at left medial temporal effect shown in Fig. 3. The contrast comparing perception of Jim versus Bob is plotted separately for Jim and Bob adaptation. In most subjects, this effect is positive after adaptation to Bob but negative after adaptation to Jim.

We next characterized the inter-subject consistency of the medial temporal effect. Therefore, for each individual subject (i.e., at the fixed effects level), we contrasted trials in which subjects reported morph faces as emotive with trials in which subjects reported morph faces as neutral, separately for emotive and neutral adaptation conditions. These data are reported in Fig. 4A for the peak voxel in the right medial temporal lobe region. For all 12 subjects, the difference between fearful and neutral perception was greater after neutral adaptation than after emotive adaptation. For eight of the subjects, emotive expression categorizations were associated with greater activation after neutral adaptation while, concomitantly, neutral expression categorizations were associated with greater activation after emotive adaptation. Thus, in most subjects, greater activity in right medial temporal cortex was associated with perception of the non-adapted expression.

For identity categorization, we tested for an interaction between adapted and perceived identity to evaluate how the difference in activity associated with perception of the two identities was modulated by adaptation to one identity or the other. Our aim was to identify regions where activity associated with perception of identity showed modulation by the adapted identity. We observed effects similar to those reported above for expression categorization: activity in bilateral anterior medial temporal structures was greater when subjects reported they perceived the non-adapted identity (Fig. 3B). In the right hemisphere, these effects were located in a cluster of 40 voxels within the ventral aspect of the anterior hippocampus (MNI coordinate of peak: 30 − 8 − 24) and additionally intersected parts of perirhinal and entorhinal cortices (Brodmann areas 36 and 28). This cluster was significant at *P* < 0.001, uncorrected, but did not survive FWE correction. In the left hemisphere (Fig. 3B), a large cluster of 300 voxels extended, posterior to anterior, along ventromedial temporal cortex, originating posteriorly in the anterior fusiform gyrus (Brodmann area 20), extending through perirhinal cortex (Brodmann area 36), and anteriorly into entorhinal cortex (Brodmann area 28) and the anterior hippocampus, where the peak voxel was located (MNI coordinate of peak: − 28 − 12 − 24). The peak voxel was significant at *P* = 0.012, FWE corrected for the whole brain. Fig. 3B shows the pattern of contrast estimates in the left medial temporal region. Again, no effects were observed in any voxels within the fusiform gyrus (posterior to the tip of the large cluster of voxels on the left) or amygdala, regions which are typically face-responsive.

Fig. 4B shows individual subject data for the voxel showing peak effects in the left medial temporal cluster. For each individual subject, we contrasted Jim categorizations versus Bob categorizations separately for each adapted identity. For all 12 subjects, adaptation to Bob resulted in increased activity in the left medial temporal lobe for perception of Jim over that of Bob. This effect was reversed after adaptation to Jim, for nine of these subjects. Thus, most subjects showed greater left medial temporal responses to the non-adapted identity category.

Although expression and identity categorization tasks were analyzed separately, they nevertheless both implicate some anterior medial temporal activity as associated with perception of non-adapted face categories, although there were some differences in the activated structures as well as in laterality. To better characterize these differences, we tested a three-way interaction, in which the sizes of the adapt × perception interactions were compared between expression and identity categorization tasks. Two small regions in right perirhinal cortex, including 13 voxels in Brodmann area 36 (MNI coordinate of peak: 28 − 14 − 28) and 34 voxels in Brodmann area 35 (MNI coordinate of peak: 20 − 32 − 22), showed a larger adapt × perception interaction for expression categorization than for identity categorization. These interactions were observed at *P* < 0.001, uncorrected, but did not survive FWE correction. No interactions with categorization task were observed in the left medial temporal cortex even at liberal uncorrected significance thresholds. Thus, the left medial temporal lobe may contribute to both aftereffects, while the right medial temporal cortex may show larger effects for expression than for identity categorization.

### fMRI responses during initial adaptation

While activity in medial temporal structures was associated with perception of different face categories in ambiguous morph faces, effects within regions known to underlie face perception – such as the amygdala and fusiform gyrus – were not observed. We therefore wished to verify whether suppression by face repetition was detectable using these data and, importantly, to evaluate whether such suppression might be measured in medial temporal regions. To achieve this, we examined activity during the initial adaptation periods — within each of which an identity (Jim or Bob) with a given expression (happy, neutral or fearful) was repeated throughout a 25-s block. Table 1 shows brain regions showing significant BOLD responses to these blocks of repeated stimuli, relative to fixation baseline. Because this contrast produced widespread effects over voxels, we chose *P* < 1 × 10^− 4^ (uncorrected) as the significance threshold to facilitate identification of separate cluster peaks.

**Fig. 5:**
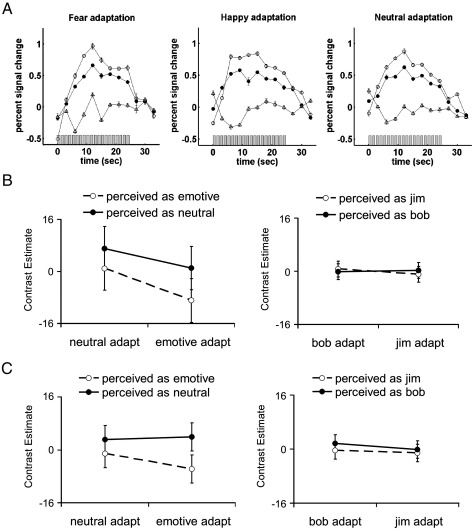
Response properties of regions activated during initial adaptation period. (A) Grey bars represent stimulus repetitions during initial adaptation period. Bilateral lingual gyrus (filled circles) and right fusiform gyrus activation (open circles) exhibit large positive responses and response reduction over stimulus repetitions. In contrast, the right medial temporal cortex effect in Fig. 3 (open triangles) shows small, nonsignificant responses. Vertical bars reflect standard errors. (B) The right fusiform gyrus activation described in (A) does not show a significant adapted category × perception category interaction in response to morph faces. (C) The right lingual gyrus activation described in (A) does not show a significant adapted category × adapted perception interaction in response to morph faces. Vertical bars reflect 90% confidence intervals of the mean contrast estimates.

As expected, viewing faces during the initial adaptation periods was associated with bilateral activation in occipital visual areas, as well as four peaks situated in the fusiform gyrus. The time courses of the regions shown in Table 1 all showed a sizable initial response, which peaked around 10 s into the block and then this response declined as faces repeated through the period. Fig. 5A shows representative time courses including the peak voxels in right lingual gyrus (MNI coordinate: 22 − 86 0) and right fusiform gyrus (MNI coordinate: 40 − 74 − 14). In contrast, medial temporal voxels show transient, low amplitude responses to faces during the adaptation period. Fig. 5A shows the time course for the peak of the right medial temporal cluster, observed when examining interaction effects during expression categorization (Fig. 3A), which shows only small responses which do not differ statistically (*P* > 0.01, uncorrected) from fixation baseline.

While the regions shown in Table 1 showed face responsivity as well as adaptation-related declines, they did not yield significant adapted category × perceived category interaction effects in response to morph faces. To more closely evaluate this null effect, we qualitatively compared the pattern of interaction effects observed in the medial temporal lobes (Fig. 3) with those of face-responsive regions activated during the initial adaptation period (Table 1). Figs. 5B and C respectively show representative interaction patterns for the same right fusiform and occipital regions described in Fig. 5A. For expression categorization, these regions show a trend towards greater activity for morph faces perceived as neutral versus emotive. However, they show no effects of adapted category or interactions. For identity categorization, these regions showed little differential responses according to adapted or perceived category.

In addition to examining face-responsive regions during the adaptation period, we also tested for regions showing differential effects of emotional expression. As regions often associated with emotion perception, such as the amygdala, did not yield significant adapted category × perceived category interaction effects in response to morph faces, we wished to have an independent verification of regions which differentiate between emotional and neutral faces within our own dataset. Those regions showing significant effects at *P* < 0.001, uncorrected, are shown in Table 2. As expected, emotional expression, relative to neutral expression, was associated with a variety of occipital visual areas in bilateral lingual gyri (which largely overlapped with the face responsive regions reported in Table 1) and a left peri-amygdala region. Also showing differential effects included regions in the left caudate and left cingulate gyri. Additionally, a cluster encompassing the right anterior hippocampus and the amygdala showed a greater effect for fearful than for happy expressions, relative to neutral expressions.

## Discussion

This study used behavioral and fMRI methods to examine the role of visual experience – in the form of face category adaptation – in facial identity and expression categorization. Behaviorally, we replicated expression aftereffects, using methods similar to previous studies (Webster et al., 2004), in which adaptation to a given facial expression increased perception of a non-adapted expression in faces morphed between the two expressions. Likewise, we observed similar effects for identity categorizations, in which adaptation to a given identity increased perception of a non-adapted identity in ambiguous faces morphed between the two identities. Although identity aftereffects have predominately been studied using adaptation to anti-faces – images computationally synthesized to bear opposing facial features to a target face (Leopold et al., 2005) – we observed identity aftereffects using a simplified design, without anti-face adaptation. Our aim was to simultaneously elicit expression and identity aftereffects using a common methodology.

We used event-related fMRI to examine how brain activity underlying perception of expression and identity categories in ambiguous morph faces is modulated by prior adaptation. This was accomplished by examining adaptive modulation of the activity distinguishing different percepts across a set of ambiguous morph faces. When examining activity in response to morph faces, we observed interactions between the adapted and perceived categories. For expression categorization, this interaction was observed in bilateral anterior ventral temporal structures including perirhinal cortex, which were more active when subjects reported that they perceived the non-adapted expression than when they reported they perceived the adapted expression (Figs. 3 and 4). For identity categorization, a comparable interaction was observed in medial temporal regions including bilateral perirhinal cortex and anterior hippocampus. After adaptation to the identity Bob, activity in these structures showed preference for perception of Jim (the non-adapted identity) over Bob. Adaptation to Jim, however, reversed this effect in most subjects (Figs. 3 and 4): anterior medial temporal structures showed preference for perception of Bob over Jim. The interaction effects obtained for expression and identity categorization conditions bear some similarity in their anatomic localization, with some overlapping of effects in bilateral perirhinal cortex. Although these data indicate a degree of variation in location and laterality, it is possible that expression and identity aftereffects share similar medial temporal-based mechanisms. The critical interaction associated with the behavioral aftereffects, however, was not observed in the fusiform gyrus or amygdala.

Adaptation-related modulation of medial temporal function is in line with a hypothesis, based largely on behavioral psychophysics, that face aftereffects are associated with advanced stages of the occipitotemporal pathway, where non-retinotopic visual representations are invariant to simple visual features and are specifically tuned to global categories. Indeed, some models of visual discrimination implicate anterior medial temporal lobes as the possible apex of the occipitotemporal visual hierarchy (Felleman and van Essen, 1991; Nakamura and Kubota, 1996), where representations of complex conjunctions of visual features are processed (Bright et al., 2005; Bussey et al., 2005; Simmons and Barsalou, 2003). Critical findings include observations that medial temporal lesions encompassing perirhinal cortex give rise to selective visual discrimination deficits in humans (Barense et al., 2005; Lee et al., 2005) and in non-human primates (Buckley et al., 2001; Buckley, 2005). Brain imaging studies show that perirhinal activations are related to the specificity of visual categorizations (Moss et al., 2005; Tyler et al., 2004). In non-human primates, many visually-responsive neurons in medial temporal structures respond selectively to complex images (Ringo, 1996), and such neurons are prevalent in perirhinal cortex (Erickson et al., 2000; Sato and Nakamura, 2003). In humans, visually-responsive neurons throughout the medial temporal lobes respond selectively to global visual categories such as faces and facial expressions (Fried et al., 1997; Kreiman et al., 2000) and show response invariance across simple visual metrics (Quiroga et al., 2005).

In contrast, earlier processing stages of the ventral pathway are associated with effects of adaptation on perception of simple visual attributes. For example, studies of the McCollough color-form contingent aftereffect report adaptive modulation of responses to test items in regions in medial lingual and fusiform gyri (Barnes et al., 1999; Humphrey et al., 1999). Also, studies of the motion-direction aftereffect report modulation of a variety of visual regions including the motion-sensitive human analogue of Area MT (Culham et al., 1999; Huk et al., 2001; Seiffert et al., 2003; Taylor et al., 2000). In an fMRI study of face viewpoint adaptation (Fang et al., 2006), responses to test items in face-selective regions including lateral occipital cortex, fusiform face area and superior temporal sulcus were sensitive to the angular disparity between adapted and test viewpoints. This finding is in line with other fMRI studies that show some degree of viewpoint-specificity in the fusiform face area and superior temporal sulcus (Andrews and Ewbank, 2004; Pourtois et al., 2005). In contrast, medial temporal neurons are responsive to individual identities across different views (Quiroga et al., 2005) and, behaviorally, identity aftereffects generalize across different views (Jiang et al., 2006). Thus, it is possible that medial temporal activity in the current experiment is associated with levels of the ventral pathway where representations are relatively viewpoint-independent.

Numerous prior studies have used a “repetition suppression” approach which focuses on localizing regions where responses to stimuli depend on the similarity to preceding “adaptation” stimuli (Grill-Spector et al., 2006). Repetition suppression studies characteristically compare conditions where stimulus dimensions are repeated against conditions where these stimulus dimensions are changing. This approach has shown, at a minimum, that fusiform gyrus activity is suppressed by identity repetition (Rotshtein et al., 2005; Winston et al., 2004). Although our overall aim was not to systematically test for category-selective repetition suppression, we nevertheless examined the time course of the BOLD response during adaptation, and observed repetition-related response reductions over a wide network of regions in the occipital and fusiform gyri (Table 1). This finding generally agrees with the repetition suppression studies described above, although our analysis is less specific and we cannot verify that this suppression is selective to identities or expressions. Many repetition suppression studies do not report medial temporal suppression in response to face repetition (Rotshtein et al., 2005; Winston et al., 2004), although some have (Henson et al., 2003; Pourtois et al., 2005). In the present experiment, medial temporal regions showed little response to blocks of repeated faces (Fig. 5A), so we could not show evidence of any repetition suppression within the medial temporal lobes. Thus, we cannot claim that medial temporal repetition suppression during adaptation can directly give rise to the interaction effects we observed in response to morph faces.

One possibility to explain the absence of medial temporal effects during initial adaptation is that as identical identities and expressions were repeated hundreds of times throughout the experiment, these medial temporal neurons were too suppressed and did not have sufficient recovery time to produce measurable BOLD responses during the initial adaptation period. Another possibility is that interpretation of apparent contrasts between the initial adaptation trials and morph trials is complicated by differing demand characteristics. During the adaptation period, the subjects repeatedly categorized the same (unambiguous) endpoint, limiting the difficulty and attentional demands required by the task. In contrast, morph faces are ambiguous and vary from trial to trial unpredictably in their content, and so responses to these faces may differ from those during the initial adaptation period due to differing attentional or stimulus processing demands. However, we note that, despite the absence of observable medial temporal suppression and despite the limited task demands, activity during the initial adaptation period was nevertheless sufficient to induce robust behavioral aftereffects.

While repetition suppression studies focus on activity modulated by stimulus manipulations (Rotshtein et al., 2005; Winston et al., 2004), our analysis of responses to morph faces instead focuses on activity modulated by the percept while holding the stimulus set constant. Because the amount of stimulus change between adaptor and morph was, on the average, the same for all conditions, we would not expect necessarily to detect the same regions which also show effects of stimulus manipulations (e.g., fusiform gyrus). Particularly, when stimulus is held constant and the percept is manipulated instead, our analysis did not reveal differential effects in the fusiform gyrus or other regions typically associated with repetition suppression. Given the modulation in these regions by stimulus manipulations, it is likely that neural populations within regions such as fusiform gyrus are engaged when assessing and detecting category-relevant information in a stimulus. When the stimuli are equated, then stimulus processing may also be equated and so equivalent fusiform activation may have resulted across conditions. Thus, adaptation aftereffects may not result from differences in how information is extracted from the stimulus, but may reflect instead the application of prior knowledge to the outcome of stimulus processing in the fusiform gyrus or other regions. Medial temporal activation may modulate the percept, then, according to recent (adapted) experience.

This poses interesting questions regarding the relative contributions of different levels of the ventral pathway to the overall perceptual aftereffect. A possibility which might explain these results is that, during the adaptation period, suppression in regions such as fusiform gyrus has cascading effects on the responsiveness of downstream medial temporal regions (Kohn, 2007). The altered responsiveness of medial temporal structures may then “passively” give rise to subsequent effects on categorization behavior. Another alternative envisages, alongside the distributed passive mechanism, a contribution from a more active mechanism localized within anterior medial temporal regions. A recent psychophysics study indeed suggests that small “passive” aftereffects induced by prior exposure and/or by subliminal adapting primes can be masked by larger “active” effects whenever subjects can consciously compare the target with a visible priming stimulus (N. van Rijsbergen, A. Jannati, and A. Treves, unpublished observation).

Medial temporal activation, particularly, perirhinal cortex is often characterized as signaling the novelty of a stimulus (Daselaar et al., 2006; Strange et al., 2005) and so has been implicated in familiarity-based memory and novelty detection (Brown and Aggleton, 2001). Novelty *detection* (per se) is not a likely possibility to explain the interactions observed in the medial temporal lobes. The morph faces were, on the average, equidistant from the adapting images and so there were no differences in novelty to detect. However, a role for relative novelty signaling of neural populations (based on a short time window of recent experience) is nevertheless directly consistent with prevailing functional accounts of perceptual aftereffects (Kohn, 2007).

For example, a popular account proposes a form of norm-based coding cast in terms of a multidimensional “face space” representation, where faces are coded as vectors describing deviations from a representation of the central tendency of experience: a norm face (Leopold et al., 2001). Recent evidence provided by psychophysics studies employing adaptation to anti-faces (Anderson and Wilson, 2005; Leopold et al., 2001, 2005; Moradi et al., 2005) suggests that adaptation shifts this norm face in the direction of the adapting stimulus in face space. The perceptual shift occurs when face vectors pointing away from the adapted category are lengthened by the shift in the norm. Thus, norm-based coding frames aftereffects as a bias towards novel percepts—those that deviate most from prior (adapted) experience. Medial temporal activity may contribute to this bias towards novel percepts. The neural mechanisms which have been proposed to underlie aftereffects also frame the categorization shift as a bias towards percepts which are novel relative to experience. For example, mechanisms proposed to underlie aftereffects include firing rate fatigue in category detecting neurons, where responses to previously experienced categories are suppressed and responses to novel stimuli can occur more easily—and so perception is shifted towards novel stimuli and away from familiar stimuli. This mechanism thus can be framed as a bias towards perception of novelty, which may be associated with medial temporal activity. Firing rate fatigue as a mechanism has been recently explored by a modeling study, which analyzed aftereffects in simple associative neural networks and found that the introduction of firing-rate fatigue was sufficient to simulate aftereffects (Menghini et al., 2007). Alternative mechanisms to firing rate fatigue can also be framed as a bias towards perception of novelty. For example, hierarchical predictive coding models posit that each visual processing stage compares bottom-up input patterns against dynamically calibrated predictions, and then signals the degree of prediction error (Friston, 2005; Rao and Ballard, 1999). As neural activity to a stimulus represents deviations from a prior expectation which is tuned to the statistics prevailing in recent experience, such activity may be interpreted as signaling novelty relative to recent experience. In this case, the bias toward novel percepts is induced by a top-down prediction signal, rather than firing rate fatigue. Predictive coding models are appealing as similar ideas have been historically proposed to explain low-level aftereffects (Andrews, 1964; Barlow and Hill, 1963; Dodwell and Humphrey, 1990; Wainwright, 1999).

More research is necessary to differentiate between adaptation models based on firing rate fatigue and models that depend on top-down predictive signals. For example, effective connectivity methods may provide a means to study how experience alters the coupling between medial temporal and other ventral stream regions. Also, inferences can be made about hierarchical processing using EEG and MEG to examine the relative timing of neural signals underlying adaptation and categorization effects associated with aftereffects. How the brain generates percepts of high-level, socially-relevant categories is an important question for research in visual neuroscience. The findings of the present experiment complement prior psychophysics data, showing that advanced stages of the ventral pathway play a role in integrating prior experience when perceiving facial identities and expressions from ambiguous stimuli. These findings also pose challenging questions regarding how the ventral pathway integrates mechanisms across various processing levels to build unified percepts of complex stimuli.

## Figures and Tables

**Table 1:** Regions showing greater activity during initial adaptation periods than fixation

Region	Brodmann areas	MNI coordinate of peak voxel			*Z*-value
*x*	*y*	*z*
*Initial adaptation period activations*					
Right fusiform gyrus	18	26	− 84	− 22	4.43
Left fusiform gyrus	18	− 28	− 84	− 20	4.52
Right fusiform gyrus	37	26	− 52	− 20	5.14*
Right fusiform gyrus	19	40	− 74	− 14	5.01*
Bilateral lingual gyrus	17	22	− 86	0	5.59*
Left middle occipital gyrus	18	− 28	− 92	8	5.0*
Left cuneus	18	− 10	− 102	8	4.85

All peak voxels significant at *P* < 1 × 10^− 4^, uncorrected, *peak voxel also *P* < 0.05, family-wise error corrected for whole brain.

**Table 2:** Differential effects of expression during initial adaptation period

Region	Brodmann areas	MNI coordinate of peak voxel			*Z*-value
*x*	*y*	*z*
*Emotive (fear and happy) adaptation > neutral adaptation*					
Left dorsal peri-amygdala	N/A	26	− 4	− 8	3.21
Left lingual gyrus	18	− 6	− 102	− 6	4.75
Right lingual gyrus	17	10	− 94	− 4	5.05*
Left lingual gyrus	17	− 12	− 104	12	4.93

*Neutral adaptation > emotive (fear and happy) adaptation*					
Cingulate gyrus	31	− 2	− 28	34	3.50

*[Fear adaptation > neutral adaptation] > [happy adaptation > neutral adaptation]*					
Right temporal pole	38	48	16	− 28	3.79
Right hippocampus/amygdala	N/A	28	− 12	− 16	3.61
Left caudate tail	N/A	− 38	− 38	2	3.58
Left cingulate gyrus	24	− 16	− 4	38	3.47
Left cingulate gyrus	31	− 18	− 44	40	3.31

*[Happy adaptation > neutral adaptation] > [fear adaptation > neutral adaptation]*					
No supra-threshold clusters					

All peak voxels significant at *P* < 0.001, uncorrected, *peak voxel also *P* < 0.05, family-wise error corrected for whole brain.

## Footnotes

1 To validate this approach, we tested a three-way interaction, in which the sizes of the adapt × perception interactions were compared between happy/neutral and fearful/neutral categorization tasks. This analysis yielded no significant effects in any ventral or medial temporal regions, even at liberal uncorrected significance thresholds.
